# Prevalence of Hypertension among Patients Attending Mobile Medical Clinics in the Philippines after Typhoon Haiyan

**DOI:** 10.1371/currents.dis.5aaeb105e840c72370e8e688835882ce

**Published:** 2016-12-20

**Authors:** Linda Meta Mobula, Mary Lou Fisher, Nathan Lau, Abi Estelle, Tom Wood, William Plyler

**Affiliations:** Johns Hopkins University, Baltimore, Maryland; Bow Valley College, Calgary, Alberta, Canada; Bow Valley College, Calgary, Alberta, Canada; Oral and Maxillofacial Surgery, UF Health Jacksonville, Jacksonville, Florida. Samaritan's Purse; Projects, Epidemiologist, Samaritan's Purse, Jonestown, Texas; Projects Department, Samaritan's Purse International Relief, Boone, North Carolina

## Abstract

Introduction: On November 8, 2013, Super Typhoon Haiyan struck the Philippines, causing a humanitarian emergency. According to the World Health Organization, non-communicable diseases (NCDs), also known as chronic diseases, are the leading cause of death and disability around the world. NCDs kill 38 million people each year.  Sixteen million NCD deaths occur before the age of 70; 82% of which occurred in low- and middle-income countries.  NCDs are further exacerbated during a crisis, and addressing them should be a concern of any medical disaster response.

Methods: We conducted a retrospective observational study to determine the prevalence of hypertension among patients seeking medical care at mobile medical clinics after Typhoon Haiyan in the Philippines.

Results: A total of 3,730 adults were evaluated at the mobile medical clinics. Analysis of the medical records revealed that the overall prevalence of hypertension among adult patients was 47%. Approximately 24% of adult females and 27% of adult males were classified with stage 2 Hypertension.

Conclusions: Evidence-based guidelines on the management of hypertension and other NCDs (diabetes mellitus, cardiovascular disease, chronic lung disease and mental health) during humanitarian emergencies are limited. Clinical care of victims of humanitarian emergencies suffering with NCDs should be a critical part of disaster relief and recovery efforts.  We therefore recommend the development of best practices and evidence based management guidelines of hypertension and other NCDs in post-disaster settings.

## Introduction

On November 8, 2013, Super Typhoon Haiyan struck the Philippines resulting in 6,000 deaths and the displacement of 3.4 million people, a humanitarian emergency. Samaritan’s Purse (SP), a faith based organization, responded to the appeal by the Philippine Department of Health for international assistance. Partnering with the Schistosomiasis Control and Research Hospital (SCRH) in Palo, Leyte, SP established and staffed a Type 1 field hospital (WHO 2013; “Minimum Standards for Foreign Medical Teams) in the parking lot of this heavily damaged hospital. In addition to providing acute medical care for the residents of the city of Palo, SP’s hospital served as a base of operations for mobile medical teams (MMTs) that deployed daily to remote locations. Essential health services were provided to residents in 40 barangays (smallest administrative division in the Philippines and is the native Filipino term for a village, district or ward) in remote and otherwise unreached areas on the islands of Leyte and Samar. Prior to Typhoon Haiyan, residents in the barangays typically accessed Barangay Health Services (BHS) for health care. After the typhoon, the death of healthcare workers and the destruction of transportation infrastructure led to the disrupted access to healthcare services.

According to the World Health Organization, non-communicable diseases (NCDs), also known as chronic diseases, are the leading cause of death and disability around the world. The four main types of non-communicable diseases are cardiovascular diseases, cancers, chronic respiratory diseases (such as chronic obstructed pulmonary disease and asthma) and diabetes; we will be referring to these four diseases in our paper. NCDs kill 38 million people each year. Sixteen million NCD deaths occur before the age of 70; 82% of these occurred in low- and middle-income countries^1^ . Healthcare systems are often disrupted in post-disaster situations, reducing access to care and medications, which can lead to exacerbation of NCDs^2^ . Literature on post-disaster morbidity and mortality among persons suffering from NCDs and management of NCD during humanitarian emergencies is limited^3^ .

In 2007, the 15th World Congress on Disaster and Emergency Medicine, held in Amsterdam, recommended that health outcome assessments of disasters, incorporate NCDs as a factor affecting the health challenges in a population^4^. Even so, humanitarian actors seldom address NCDs specifically or assess their impact. This may be because the acute needs of those with traumatic injuries consume available resources, and acute medical issues in mass casualty events capture the focus of responders. Disregard of the medical needs of persons with NCDs can add to the toll of suffering resultant from the catastrophe. The lack of literature on NCDs in disasters points to a need for further investigation into the care of those with NCDs during humanitarian emergencies^5^.

More information on the extent and nature of the issue could facilitate better planning and preparation by humanitarian agencies seeking to address the overall morbidity and mortality associated with traumatic events. This report presents a post disaster evaluation of the burden of hypertension among victims of Super Typhoon Haiyan in the Philippines.

## Methods

A retrospective observational study was completed in order to determine the prevalence of hypertension among patients attending clinics conducted by mobile medical teams (MMTs) in 40 barangays located in central Leyte and Western and Eastern Samar, island provinces within the Philippine Archipelago. Medical records were kept for all patients. Patient evaluations included a history and physical, during which vital signs were measured and recorded. Blood pressure readings were taken using sphygmomanometers and stethoscopes. Results were recorded in patient medical records as systolic/diastolic pressure expressed as millimeters of mercury (mmHg). These results were then entered into an electronic patient database.

Causes of morbidity were documented and submitted daily to the Department of Health of the Philippines utilizing that department’s “Surveillance in Post Extreme Emergencies and Disasters” (S.P.E.E.D.) forms.

The Sixth Report of the Joint National Committee on Prevention, Detection, Evaluation, and Treatment of High Blood Pressure (JNC VI) defined and classified hypertension in adults, as systolic BP ≥140 mm Hg and diastolic BP ≥ 90 mm Hg^6^. Stage 1 Hypertension is defined as a systolic pressure ranging from 140 to 159 mm Hg or a diastolic pressure ranging from 90 to 99 mm Hg. Stage 2 Hypertension is defined as a systolic pressure of 160 mm Hg or higher, or a diastolic pressure of 100 mm Hg or higher. For the purpose of this study only adult patients, those 18 years old or older, were included.

Ethical approval was obtained from the Philippines Ministry of Health, Regional Office VIII, Leyte, Philippines.

## Results

The top three diagnoses among adult patients were: a) acute respiratory illness (with or without bronchospasm and associated complications), b) hypertension (HTN), and c) puncture wounds requiring tetanus immunization as well as those complicated by infection. Clinical care was provided to a total of 3,730 adults, including 1,020 males and 2,710 females. Of these, blood pressure (BP) results were available on 986 males and 2,647 females for a total of 3,633 adults. The male to female patient ratio approximated 1:3. The mean age of adult patients was 50.4 ± 17.1 years (Table 1).

**Figure:**
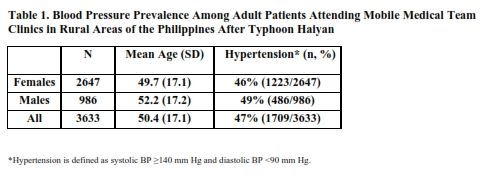

**Figure 1:**
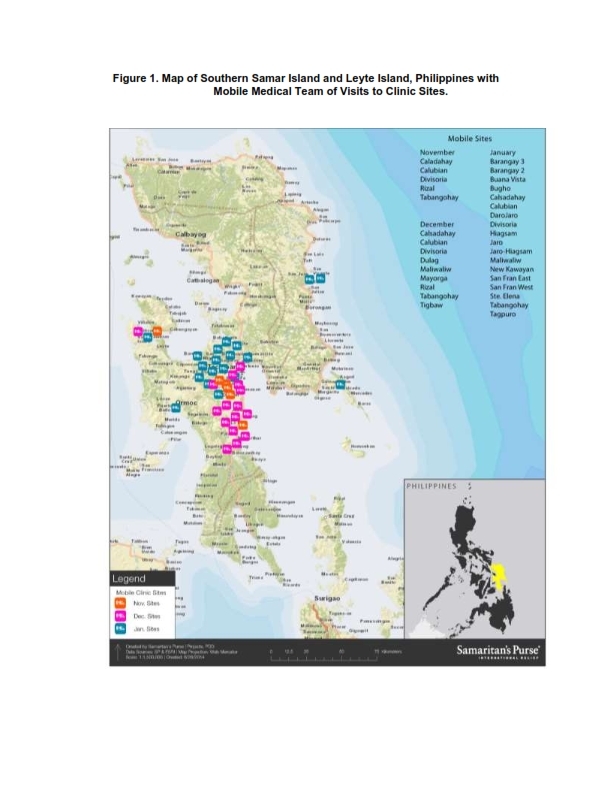

Analysis of the medical records from all clinic visits revealed that the overall prevalence of hypertension among adult MMT clinic patients was 47% (47 per 100 individuals). In 34 out of the 40 barangays more than 40% of patients had BP readings consistent with a diagnosis of hypertension. Hypertension was especially prevalent in the following barangays (district): New Kawayan, Rizal Dagami, Guinan Cogdara-o, Basay, Bugho, Basey Balud, Roxas and Tigbaw (Figure 1).

Further analysis of BP readings from adult patients seeking care through an MMT clinic revealed that 22.2% exhibited stage 1 Hypertension (S1HTN) and 24.9% presented with stage 2 Hypertension (S2HTN). Stratified by gender, 22.7% of males presented with S1HTN and 26.6% had blood pressure readings consistent with S2HTN. Among female patients 22% had S1HTN while 24.2% exhibited S2HTN (Table 2).

**Figure:**
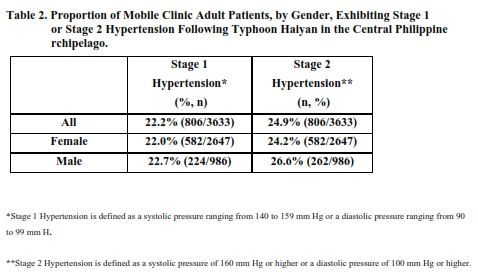

## Discussion

A retrospective review of the medical records of MMT clinic adult patients revealed that the most common reason given for their seeking care was for treatment of acute respiratory illness followed by hypertension. Our study had several limitations. We did not collect socio-demographic data such as obesity, smoking status, hypercholesteremia, etc. Second, we were not able to evaluate end-organ damage.

There were fewer adult males compared to adult females attending MMT clinics. The need to rebuild and rehabilitate devastated infrastructure, as well as earn a wage to sustain their families, made it difficult for working male adults to access medical care. Among the males seeking care through an MMT clinic, a high percentage exhibited either Stage 1 or Stage 2 hypertension, although hypertension was rarely their primary reason for seeking care.

We selected antihypertensive therapy from Standard Treatment Guidelines used in the Philippines, which were administered at conservative doses to achieve blood pressure control. Anticipating that the Filipino Department of Health would be recovering for a protracted period, temporary measures were implemented to bridge gap for hypertension management. A 10 to 30 day supply of medications was dispensed in anticipation of restoration of health services. Patients with a previous diagnosis of hypertension often reported decreased access to medicine as a result of the typhoon. With approval by the Filipino Department of Health and local leaders, SP attempted to bridge the gap in the acute phase post-disaster by distributing of sphygmomanometers and related equipment, along with antihypertensive medications, for future use by local healthcare workers and rural health units. It is recommended that all NGOs that have a health response during a disaster work closely with the Ministry of Health to ensure the sustainability of chronic disease management during the recovery phase.

Patients with hypertensive urgency and who were symptomatic (neurological changes, associated headache, chest pain, etc.) were referred to a functioning hospital that had not been seriously impacted by the typhoon. When warranted, assistance with transportation was provided to the closest health care facility. Interim medical supply chains may be required to bridge healthcare needs while medical infrastructure is reestablished. With that in mind, it is paramount that humanitarian actors work in coordination to extend and maximize temporary availability of medical supplies and services to address NCDs.

The prevalence of hypertension among this patient population was likely impacted by diarrheal diseases and dehydration secondary to the lack of access to potable clean water frequently seen in the aftermath of a disaster. In patients with a known diagnosis, the most common reason for seeking care was acute respiratory illness.

Previous studies in post-disaster settings have reported that blood pressure in some individuals, confounded by anxiety and post-traumatic stress, may remain abnormally elevated for months^7^^,^ 8 ^,^ 9 ^,^ 10. While it is possible that patients with a known history of hypertension may be slightly over represented in relief health services to obtain medication refills, the overall experience in the MMT clinics did not support this as the motivation for care-seeking behavior after Typhoon Haiyan. As previously noted there were fewer adult males compared to adult females attending MMT clinics. The need to rebuild and rehabilitate devastated infrastructure, as well as earn a wage to sustain their families, made it difficult for working male adults to access medical care. Among the males seeking care through an MMT clinic, a high percentage exhibited either Stage 1 or Stage 2 hypertension.

Preyson 3, a prospective, multi-staged, stratified nationwide survey on hypertension found that the prevalence of hypertension in the Philippines was 28%^11^. Among those with NCDs, the majority of patients presented with elevated blood pressure. The prevalence of hypertension in a population cluster sample of displaced persons in shelters after hurricane Karina was found to be 34%, while the prevalence of hypertension among victims of the Wenchuan earthquake in China was 24%^12^^,^ 13. The prevalence of hypertension in China in this population was similar to the prevalence prior to the earthquake (27%). The population of displaced person after hurricane Katrina likely had chronic hypertension and it is not clear whether the prevalence of hypertension increased in the aftermath of Katrina.

A surprising 47% of the adult population evaluated at our MMT clinics exhibited evidence of elevated blood pressure; though it’s not clear if this was secondary to chronic disease or just elevated due to stress in the post-disaster setting. Unfortunately, patient reporting of pre-disaster medical history was not always reliable. Patients with a previous diagnosis of hypertension often reported decreased access to medicine as a result of the typhoon as a reason for care seeking. Had the disaster happened in a community with more complete medical records, we would be able to determine more accurately what percentage of the hypertension or elevation in pressure was due to stress secondary to disaster-related factors.

Typically, hypertension guidelines recommend that systolic and diastolic blood pressure be reduced to values less than 140/90 mm Hg and that control for patients with hypertensive urgency be achieved conservatively over hours or days^14^. Some studies suggest that management of hypertensive urgency (even in the short term) could be clinically beneficial. Unaddressed hypertensive urgency not only increases the risk of cardiovascular and cerebrovascular events, but also the progression towards end organ damage^15^.

There is evidence to suggest that tight control of hypertension is beneficial and can reduce cardiovascular outcomes^16^. In the Cardio-Sis trial, where patients were randomized to tight versus usual control, it was found that new-onset atrial fibrillation and coronary revascularization occurred less in the tight-control group. However, the incidence of myocardial infarction, admission for heart failure, stroke, transient ischemic attack, and all-cause mortality was low and did not differ between the groups^16^. It is important to note that the follow-up period was two years for this study and therefore might not be applicable to this particular setting. It is not clear if treating hypertension for a few weeks will result in significant changes in cardiovascular outcomes in the long run. In protracted conflicts such as the Syrian crisis, these results would perhaps be more relevant.

In a post-hoc analysis of the Cardio-Sis trial, the risk of composite cardiovascular end point was higher in the group with cardiovascular disease at baseline than in the group without^17^. The risk of cardiovascular outcomes was also lower in patients assigned to the tight control than in those in the standard BP control group. The benefits of controlling hypertension with respect to stroke, renal, and cardiovascular disease complications led international guidelines to recommend reduction of BP to <140/90 mm Hg for uncomplicated hypertension or <130/80 mm Hg for subjects with co-morbid kidney or cardiovascular disease^17^. However, it is less clear whether adverse outcomes will occur in the setting of non-compliance in the short-term, or whether tight control is beneficial in a post-disaster setting.

Several studies have shown an increase in mortality secondary to cardiac causes following an earthquake^18^^,^ 19 ^,^ 20 ^,^ 21 ^,^ 22 ^,^ 23. One such study conducted in Japan following the Hanshin Awaji earthquake in 1995, postulates that increased hypertension contributed to a threefold increase in myocardial infarction among populations in close proximity to the earthquake, particularly in women, and a near doubling in the frequency of strokes^23^. Hypertension is known as the single most important risk factor for ischemic strokes. It is seen in 75% of patients with acute ischemic stroke, in 80% of patients with acute intracerebral hemorrhages, and is independently associated with poor functional outcome^24^. Unaddressed hypertensive urgency not only increases the risk of cardiovascular and cerebrovascular events, but also the progression towards end-organ damage.

It would therefore be important to have cohort studies in place that would be able to track outcomes following a disaster. The importance of prospective cohort studies that are able to evaluate both short and long-term outcomes following a disaster is paramount. In hypertensive urgency (≥180/≥110 mm Hg), the absence of clear clinical events at the time of a disaster (such as stroke, renal failure, or a myocardial infarction) makes it difficult to understand the impact of treatment interruption in the short-term.

Our study is relevant as it contributes to the body of literature on the burden of hypertension in the post-disaster period. Further studies are needed to determine blood pressure targets for victims of disasters who do not have access to treatment in the aftermath of a disaster. Additionally, it is important to understand whether patients that are at high risk of developing negative outcomes, such as those with concomitant kidney disease and cardiovascular disease, require more tight control of their blood pressure. It is important to develop better guidelines that provide more detailed recommendations on blood pressure targets, on the optimal use of drugs to treat hypertension (especially when one does not have appropriate follow-up) and groups that require tight control.

It is recommended that other NGOs that have a health response during a disaster disasters work closely with Ministries of Health to ensure the sustainability of chronic disease management during the recovery phase. Without this partnership, access to medicines and services required to manage NCDs will remain a challenge.

## Conclusions

Better planning and preparation by humanitarian actors seeking to decrease the overall morbidity and mortality associated with disasters should include treatment of NCDs. There is limited evidence regarding the optimal management of hypertension in disaster settings. Clinical care of patients with hypertension and other NCDs is an important part of disaster relief and recovery. We recommend future studies to determine best practices and evidence-based management of other NCDs (such as diabetes mellitus, cardiovascular disease, chronic lung disease, mental health etc.) in post-disaster settings.

## Competing Interests

The authors have declared that no competing interests exist.

## Data Availability

Data are available in Figshare at https://figshare.com/s/adcd8bc96bd0164cc107

## Corresponding Author

Dr. Linda Mobula: mmobula1@jhmi.edu
